# Gender differences in metabolic syndrome components among the Korean 66-year-old population with metabolic syndrome

**DOI:** 10.1186/s12877-016-0202-9

**Published:** 2016-01-23

**Authors:** Sangjin Lee, Young Ko, Chanyeong Kwak, Eun-shil Yim

**Affiliations:** 1 Office for Planning and Coordination, Division of Planning and Coordination, Ministry of Health & Welfare, 13, Doum 4-ro, Sejong-si, 339-012 Republic of Korea; 2 College of Nursing, Gachon University, 191 Hambakmoeiro, Yeonsu-Gu Incheon, 406-799 Republic of Korea; 3 School of Nursing, Hallym University, 1 Hallymdaehak-gil, Chuncheon, Gangwon-do 200-702 South Korea; 4 Department of Nursing, Daegu Health College, Youngsong-ro, Buk-gu Daegu, 702-722 Republic of Korea

**Keywords:** Metabolic syndrome, Korea, Old adult, Gender, Life transition, Cardiovascular disease

## Abstract

**Background:**

Gender is thought to be an important factor in metabolic syndrome and its outcomes. Despite a number of studies that have demonstrated differences in metabolism and its components that are dependent on gender, limited information about gender differences on the characteristics of metabolic syndrome and its components is available regarding the Korean old adult population. This study aimed to identify gender differences in characteristics of the metabolic syndrome and other risk factors for cardiovascular disease.

**Methods:**

Secondary analysis of data from a nationwide cross-sectional survey for health examination at the time of transitioning from midlife to old age was performed. Multiple logistic regression models were used to estimate adjusted odds ratios and 95 % confidence intervals for gender differences among the Korean 66-year-old population with metabolic syndrome.

**Results:**

Gender differences in metabolic syndrome components that contributed to the diagnosis of metabolic syndrome were identified. In males, the most common component was high blood sugar levels (87.5 %), followed by elevated triglyceride levels (83.5 %) and high blood pressure (83.1 %). In females, the most commonly identified component was elevated triglyceride levels (79.0 %), followed by high blood sugar levels (78.6 %) and high blood pressure (78.5 %). Gender differences for other risk factors for cardiovascular disease, including family history, health habits, and body mass index were observed.

**Conclusions:**

Gender-specific public health policies and management strategies to prevent cardiovascular disease among the older adult population should be developed for Koreans undergoing the physiological transition to old age.

## Background

Metabolic syndrome (MetS) is generally thought to be a consequence of social and environmental changes related to urbanized living conditions, high-caloric food intake, and sedentary lifestyle [1]. MetS is one of the most common diseases in the elderly [2–7] and accounts for 25 % of the global burden of disease in adults [8]. In Korea, cardiovascular disease (CVD) is the second-leading cause of death [9]. Korean studies have shown men and women with MetS have a 48 % greater risk of CVD and 60 % greater risk of CVD mortality compared with those without MetS [10].

MetS is referred to as the group of cardiovascular risk factors that result from insulin resistance and include hyperglycemia, dyslipidemia, hypertension, and central adiposity [9]. MetS can precede overt diabetes but is also related to the development and prognosis of CVD [11, 12]. Due to various definitions of MetS and unexplained pathophysiology, there is medical controversy surrounding MetS [13]. However, it is well established that MetS is strongly related with increased incidence of CVD [8, 11, 12].

Studies have shown that MetS prevalence differs according to gender, age, and country [2–7, 14, 15]. Using the National Cholesterol Education Program’s (NCEP) definition of MetS, age-adjusted prevalence of MetS among adults over 20 years of age is estimated at 31.3 % in Korea [4], which is similar to estimates in the US [2]. The prevalence of MetS has been increasing [4, 14] over the last decades; this increase has been attributed to an increased incidence of dyslipidemia and abdominal obesity in Korean adults [4, 14]. Among old adults, MetS prevalence has been reported to vary from 20 to 60 %, with higher prevalence in females than males in several studies [3, 5–7, 15]. However, limited information is available regarding gender differences in MetS characteristics and components among older Koreans.

Since 2007 in Korea, the national health screening program at the time of life transition from midlife to old age has been performed in the 66-year-old population; this has not been performed to identify disease but to plan preventive health management [16]. The prevalence rate of CVD among the older adult population is increasing in Korea [6], which suggests that it is important that policymakers be aware of the severity of this issue, allowing for the preparation of guidelines and management measures.

Thus, this study aimed to determine gender differences in relation to characteristics of MetS and other CVD risk factors using the data acquired during the health screening of the 66-year-old Korean population. This study provides important gender-specific information about the management of MetS to prevent CVD in Koreans transitioning to old age.

## Methods

### Study design

Secondary analysis of data obtained using a nationwide cross-sectional survey was performed. The survey was performed by the National Health Insurance Corporation (NHIC) and was used for health evaluation in transition periods of life in 2008 [17].

### Subjects and settings

A total of 446,813 individuals were included in the target population (age = 66 years) in South Korea. Of these, 58.5 % (261,559 individuals) received a routine health examination between January and December 2008. The data of 103,763 (43,129 males, 60,634 females) individuals with MetS were used in the secondary analysis.

### Measurements

#### Definition of MetS

Individuals with MetS were identified using the definition of the American Heart Association/National Heart Lung, and Blood Institute (AHA/NHLBI) [18]. The NCEP was adopted to diagnose MetS in this study (Table 1). To account for ethnic differences in body shape, waist circumference standards defined by the World Health Organization for obesity in Asian-Pacific regions were used [19]. Individuals who demonstrated normal findings as a result of medical treatment were also included in the analysis.

**Table 1:** Criteria of metabolic syndrome by the National Cholesterol Education Program

Component	Criteria
Central obesity	Waist measurement >90 cm (male) or >80 cm (female)
Low HDL cholesterol	HDL-C <40 mg/dL (male) or <50 mg/dL (female)
High blood pressure	BP >130/85 mmHg
Hypertriglyceridemia	TG >150 mg/dL
Elevated blood sugar	Fasting blood sugar >100 mg/dL

*HDL* high density lipoprotein, *BP* blood pressure, *TG* triglyceride

#### Family history, obesity status, and health behaviors

The data were collected in the first health examination of the national health-screening program using questionnaires, measurements, and laboratory tests. Trained testers measured height, weight, waist circumference (WC), and blood pressure (BP). Body mass index (BMI) was calculated using height and weight, and subjects were categorized as underweight (BMI <18.5 kg/m^2^), normal (BMI of 18.5–25 kg/m^2^), overweight (BMI of 25–29 kg/m^2^), or obese (BMI ≥30 kg/m^2^) [20]. Blood tests for fasting blood sugar (BS), high-density lipoprotein cholesterol (HDL-C), and triglycerides (TG) levels were performed. A questionnaire was used to obtain family history of diseases, including stroke, CVD, hypertension, and diabetes, and information about health behaviors, including smoking, alcohol consumption, and exercise habits.

Smoking behaviors were assessed using two questions: subjects were asked (1) whether they had smoked more than five packs of cigarettes in their lifetime and (2) if they had, whether they currently smoked. Smoking status was categorized into “non-smoker,” “past smoker,” or “current smoker.” Alcohol consumption was defined as the average frequency of alcohol consumption per week; subjects were categorized as “no alcohol consumption,” “1–3 times per week,” or “more than three times per week.” Physical activity level was defined according to the number of days of aerobic physical activity per week; subjects were categorized as either “lower moderate-intensity physical activity” or “higher moderate-intensity physical activity.” Subjects who performed 30 min of moderate-intensity aerobic activity at least 5 days per week or 20 min of vigorous aerobic activity at least 3 days per week were classified as “higher moderate-intensity physical activity.”

### Ethical considerations

The national health-screening program at life transition is performed annually in the 40-year-old and 66-year-old populations and is funded by health care insurance. First, inquiry sheets for the first health examination were sent by mail to all potential participants in Korea. Recipients were able to obtain a health examination within the relevant year at any center. When the health examination was performed, recipients were asked if they allowed for the use of their information for statistics and/or study purposes. The written informed consent forms were obtained from each recipient before the health examination. The data used in this study were provided by NHIC according to its own regulation. As the internal information disclosure regulation of NHIC, any information that allowed for identification of an individual was eliminated before analyses. This study used only de-identified pre-existing data with no subject contact, and permission to conduct the present study was approved by the ethics review board of the University (HIRB-2014-53) with which the researcher was affiliated.

### Statistical analysis

To analyze the data, frequencies were calculated for categorical variables; means, standard deviations, and ranges were calculated for continuous variables. To explore relationships between family history, health behaviors, obesity status, components of MetS diagnosis, combinations of MetS components, and gender, chi-square tests were performed to compare categorical variables, and *t*-tests were performed to compare continuous variables.

## Results and discussion

### Results

#### Characteristics of 66-year-old adults with MetS

The family histories and health behaviors of the 103,763 MetS subjects included in the analysis are shown in Table 2. There were significant differences between males and females for most variables. Family history of disease differed significantly between males and females. Specifically, family history of CVD was 43.5 % for males and 38.8 % for females (*p* <0.0001), and family history of diabetes mellitus was 40.7 % for males and 36.2 % for females (*p* <0.0001).

**Table 2:** Family history of disease, health behaviors, and obesity status by gender

	Male (*n* = 43,129)	Female (*n* = 60,634)	*p-*value
Family history of cardiovascular disease			<0.0001
Yes	18,777 (43.5 %)	23,553 (38.8 %)	
No	24,352 (56.5 %)	37,081 (61.2 %)	
Family history of diabetes			<0.0001
Yes	17,570 (40.7 %)	21,949 (36.2 %)	
No	25,559 (59.3 %)	38,685 (63.8 %)	
Smoking status			<0.0001
Non-smoker	18,029 (41.8 %)	58,633 (96.7 %)	
Past smoker	13,367 (31.0 %)	550 (0.9 %)	
Current smoker	11,733 (27.2 %)	1,451 (2.4 %)	
Alcohol consumption			
No alcohol consumption	19,940 (46.2 %)	55,282 (91.2 %)	<0.0001
1–3 times per week	11,794 (27.3 %)	4,297 (7.1 %)	
≥3 times per week	11,395 (26.4 %)	1,055 (1.7 %)	
Physical activity			<0.0001
Lower moderate-intensity	38,707 (89.7 %)	57,164 (94.3 %)	
Higher moderate-intensity	4,422 (10.3 %)	3,470 (5.7 %)	
Obesity status			<0.0001
Underweight (<18.5 kg/m^2^)	658 (1.5 %)	500 (0.8 %)	
Normal (18.5–25 kg/m^2^)	21,006 (48.7 %)	25,333 (41.8 %)	
Overweight (25–29 kg/m^2^)	19,819 (46.0 %)	29,725 (49.0 %)	
Obese (>30 kg/m^2^)	1,646 (3.8 %)	2,076 (8.4 %)	
	43,129 (100.0 %)		

Data are presented as number (percentage)

*n* number

Health behaviors differed significantly between males and females. The percentage of current smokers was significantly higher for males (27.2 %) than for females (2.4 %; *p* <0.0001). The percentage of males (26.4 %) who drank alcohol more than three times per week was significantly higher than the percentage of females (1.7 %; *p <*0.0001). In addition, the percentage of males (10.3 %) who performed higher moderate-intensity physical activity was significantly higher than the percentage of females (5.7 %; *p* <0.0001). The proportion of males and females within each BMI group also differed. Specifically, the percentage of females in the overweight and obesity groups was higher than that of males, and the percentage of individuals in the underweight group was higher in males (1.5 %) than in females (0.8 %; *p* <0.0001).

#### Gender differences in MetS components

Comparisons of the five components of MetS by gender are shown in Table 3. Males and females differed significantly in the rates of all components of MetS: abdominal obesity (males: 54.4 %, females: 60.8 %; *p* <0.0001), low HDL-C levels (males: 31.3 %, females: 59.0 %; *p* <0.0001), high TG levels (males: 83.5 %, females: 79.3 %; *p* <0.0001), high BP (males: 83.1 %, females: 78.5 %; *p* <0.0001), and elevated BS levels (males: 87.5 %, females: 78.6 %; *p* <0.0001). There was a statistical difference in the number of abnormalities between males and females, with females likely to have more abnormalities than males.

**Table 3:** Anthropometric variables and components of metabolic syndrome of subjects with metabolic syndrome categorized by gender

	Male (*n* = 43,129)		Female (*n* = 60,634)		
	Mean ± SD	*n* (%)	Mean ± SD	*n* (%)	*p*-value
Waist circumference (cm)					<0.0001
Normal	82.30 ± 5.39	19,655 (45.6 %)	78.19 ± 4.89	23,742 (39.2 %)	
Obese^a^	94.78 ± 8.17	23,474 (54.4 %)	90.89 ± 10.18	36,892 (60.8 %)	
HDL cholesterol (mg/dL)					<0.0001
Normal	65.54 ± 73.14	29,641 (68.7 %)	82.76 ± 97.43	24,858 (41.0 %)	
Low^b^	34.23 ± 4.16	13,488 (31.3 %)	41.54 ± 5.74	35,776 (59.0 %)	
Triglyceride (mg/dL)					<0.0001
Normal	105.29 ± 27.39	7,133 (16.5 %)	105.79 ± 27.04	12,751 (21.0 %)	
High^c^	195.05 ± 111.05	35,996 (83.5 %)	192.91 ± 100.46	47,883 (79.0 %)	
Blood pressure (mmHg)					<0.0001
Normal	126.35 ± 12.50/75.47 ± 6.60	7,277 (16.9 %)	126.08 ± 13.09/74.82 ± 6.86	13,011 (21.5 %)	
High^d^	136.43 ± 17.05/83.16 ± 10.68	35,852 (83.1 %)	136.31 ± 6.86/82.49 ± 10.86	47,623 (78.5 %)	
Blood sugar (mg/dL)					
Normal	89.18 ± 7.54	5,404 (12.5 %)	88.94 ± 7.37	13,002 (21.4 %)	<0.0001
Elevated^e^	116.18 ± 34.99	37,725 (87.5 %)	113.56 ± 32.60	47,632 (78.6 %)	
Number of abnormalities					
3		28,221 (65.4 %)		33,048 (54.5 %)	
4		12,668 (29.4 %)		21,268 (35.1 %)	
5		2,240 (5.2 %)		6,318 (10.4 %)	

Data are presented as mean ± standard deviation or as number (percentage)

^a^ Obese is defined as waist circumference >90 cm (male) or >80 cm (female). ^b^ Low HDL cholesterol is defined as HDL <40 mg/dL (male) or <50 mg/dL (female). ^c^ High triglyceride is defined as triglyceride >150 mg/dL. ^d^ High blood pressure is defined as systolic/diastolic blood pressure >130/85 mmHg. ^e^ Elevated blood sugar is defined as fasting blood sugar >100 mg/dL

*n* number, *HDL* high-density lipoprotein

The prevalence of having three, four, or five MetS components is presented in Table 4. The most prevalent combination of MetS components for both genders was the clustering of TG, BP, and BS. However, the clustering of high TG levels, high BP, and elevated BS levels was more prevalent for males (30.8 %) than females (14.5 %; *p* <0.0001). All combinations of MetS components had a different prevalence among males and females (*p* <0.0001).

**Table 4:** Prevalence of metabolic syndrome components combination by gender

Combination	Male (*n* = 43,129)	Female (*n* = 60,634)	*p*-value
	*n* (%)	*n* (%)	
Three components			
TG, BP, BS	13,286 (30.8 %)	8,780 (14.5 %)	<0.0001
WC, BP, BS	4,039 (9.4 %)	4,169 (6.9 %)	<0.0001
WC, TG, BS	3,082 (7.1 %)	2,689 (4.4 %)	<0.0001
HDL-C, TG, BS	1,508 (3.5 %)	4,811 (5.4 %)	<0.0001
WC, TG, BP	2,131 (4.9 %)	2,480 (4.1 %)	<0.0001
HDL-C, TG, BP	1,054 (2.4 %)	3,066 (5.1 %)	<0.0001
HDL-C, BP, BS	1,059 (2.5 %)	2,294 (3.8 %)	<0.0001
WC, HDL-C, TG	791 (1.8 %)	2,351 (3.9 %)	<0.0001
WC, HDL-C, BP	602 (1.4 %)	2,251 (3.7 %)	<0.0001
WC, HDL-C, BS	668 (1.5 %)	1,665 (2.7 %)	<0.0001
Four components			
WC, HDL-C, TG, BS	7,103 (16.5 %)	6,740 (11.1 %)	<0.0001
WC, HDL-C, TG, BP	2,748 (6.4 %)	6,299 (10.4 %)	<0.0001
WC, TG, BP, BS	1,228 (2.8 %)	3,003 (5.0 %)	<0.0001
WC, HDL-C, BP, BS	764 (1.8 %)	2,372 (3.9 %)	<0.0001
HDL-C, TG, BP, BS	825 (1.9 %)	2,854 (4.7 %)	<0.0001
Five components			
WC, HDL-C, TG, BP, BS	2,240 (5.2 %)	6,318 (10.4 %)	<0.0001

*WC* obsessed waist circumstance, *HDL-C* low HDL cholesterol, *TG* high triglyceride, *BP* high blood pressure, *BS* elevated blood sugar

### Discussion

This study showed that gender differences in the components of MetS contributed to the diagnosis of MetS in individuals at the time of transitioning from midlife to old age. The main gender differences observed were as follows: a higher prevalence of abdominal obesity and low HDL-C in females than in males and a higher prevalence of high TG level, high BP, and elevated BS levels in males than in females.

Most studies have reported the presence of gender differences in the prevalence of MetS components [5–7, 14, 21, 22]. However, the gender-specific differences in characteristics are not consistent across studies. For example, studies performed in China found that the prevalence of TG, HDL-C, and WC among males is higher than the prevalence in males among Chinese aged 60 years or older [22]. In Turkey, it has been shown that older adult females had higher systolic BP, larger WC, and lower HDL-C than older males [7]. One explanation for these inconsistent findings regarding gender difference could be age differences in the study populations. Gender differences are thought to be further modified by age [5, 14]. In Korea, the prevalence of TG and BP was higher in females than in males aged 60 years or older, while the prevalence of TG and BP was higher in males than in females aged 20–59 [14]. Another possible explanation of gender differences may be explained by confounding factors such as genetic traits [15], dietary habits [23, 24], level of physical activity [6], and socioeconomic status [25].

Similar to what has been observed in other studies of MetS for older adults [2, 7, 26, 27], we observed that among 66-year-old females with MetS, increased WC and low HDL-C levels were large contributors to MetS. In a previous study, the prevalence of increased WC and low HDL-C was higher in females than in males, and the prevalence of WC and HDL-C increased with age in Korea [14]. It is likely that these results are related to the relatively greater increase in visceral abdominal fat (VAT) and changes to blood lipid concentrations observed following menopause [27–29], suggesting that addressing health issues of women following menopause is important. Women who become postmenopausal had a significantly increased VAT [28, 29]. VAT accumulation is generally accompanied by insulin resistance, increased free fatty acid concentration, and secretion of apolipoprotein B-containing particles, leading to hypertriglyceridemia and increased hepatic lipase activity. These lipid changes (increased TG and low HDL-C) might contribute to the number of women meeting a diagnosis of MetS [30]. Therefore, health management during menopausal periods should be emphasized to prevent MetS in older women.

While there are similar predisposing factors in prevalence of MetS across many other countries [2, 7], the study also found different factors in explaining gender difference. The current results show that elevated TG level (79.0 %) was the most commonly observed component in females, followed by elevated BS levels (78.6 %) and high BP (78.5 %). In males, the most commonly observed MetS component was elevated BS levels (87.5 %), followed by elevated TG level (83.5 %) and high BP (83.1 %). In the MetS subjects included in this study, the majority (male: 40.7 %; female: 34.6 %) had three components of MetS.

High BP was prevalent for both genders in this study, though slightly more so for males. It has been shown previously that for males and females in Japan, BP was the most common risk factor for MetS in individuals with a single risk factor [5]. Similarly, in studies examining MetS patients in Turkey and China, the most frequently observed component of MetS was high BP [7, 22].

In this study, the percentage of MetS patients with elevated fasting BS levels was higher for males than females, similar to observations in the US [2], Japan [5], Turkey [7], and Germany [31]. In addition, the prevalence of elevated fasting BS was higher than in other countries [2, 7, 14, 31]. This observation is likely related to sex hormones. It is reported that low sex hormone-binding globulin that results in high free androgen concentrations and low testosterone concentrations are correlated with abnormal glucose tolerance and TG after adjusting for age and BMI in Korean males [32]. However, this result should be interpreted with caution since this study only used impaired fasting glucose (IFG) level as the criterion for MetS diagnosis. The prevalence of IFG and impaired glucose tolerance (IGT) differs between genders because of differences in lean muscle mass, visceral adiposity, and the influence of aging and menopausal transition [30]. It has been reported that IFG is more common in men than in women across nearly all age groups, while IGT is higher in women, except among those over the age of 60 years in Asian populations [32]. Therefore, the prevalence of abnormal glucose homeostasis in females could be underestimated in this study. Underdiagnosis of diabetes in Korea was reported for non-obese old adults when only fasting BS was used [33]. Few studies have been conducted to identify the gender difference in both the diagnostic method of IFT and IGT in an old population. Therefore, further studies are recommended in this matter.

Surprisingly, the prevalence of high TG levels was high for both genders, though slightly more so for males. This was not observed in previous studies conducted in Japan, Turkey, and Canada [5–7]. More than half of Koreans were reported to consume diets with a carbohydrate energy ratio above 70 % [23, 24]. Binge drinking frequency has also been reported to be associated with high TG, high BS, high BP, and abdominal obesity in men and with high BS and high BP in women in Korea [34]. The prevalence of hypertriglyceridemia may be higher than in other countries due to a high carbohydrate diet (particularly if they are refined carbohydrates) and excess alcohol consumption [23, 34]. In addition, Asian adults tend to have higher insulin resistance than people in the West [35, 36]. Insulin resistance is commonly associated with elevated serum TG levels [37], which accounts for the prevalence of high TG levels in MetS patients of both genders. TG levels could be used as a sensitive marker of the incidence of MetS in Koreans. Several studies support that TG concentration is an independent risk factor in the development of CVD [38, 39]. Therefore, routine monitoring of TG levels and changing unhealthy behaviors—such as diets high in refined carbohydrate, sedentary lifestyles, and excess alcohol consumption—are important for preventing CVD in older adults with MetS.

The study also found that elevated TG, BP, and BS were prevalent components of MetS in both genders, especially males. It has been reported previously that fasting BS levels are the highest predictive factor for CVD and diabetes with MetS in elderly Koreans [40]. In addition, it has been reported that BP has a greater impact on CVD mortality than do other components of MetS in Koreans, especially in females [41]. Therefore, we suggest the introduction of public health programs aimed at improving TG, BS, and BP control for older adults with MetS to reduce the incidence of CVD in Korea.

In Korea, older adult females have a higher prevalence of MetS than older adult males [14, 15]. In this study, MetS was more severe in females than in males, which is consistent with previous findings [7, 22]. The prevalence of MetS in Korean females increases in adults 60 years of age or older [14]. The number of MetS components is linearly associated with the risk of CVD, stroke, and diabetes [10]. Additional consideration is required for older females with MetS.

There were gender differences for other risk factors of CVD observed in this study. Males with MetS had worse smoking habits than females, while females with MetS had lower physical activity levels and higher BMIs than males, which has also been observed previously in Korea and the UK [14, 42]. Therefore, gender-specific programs designed to change health behaviors and prevent CVD events should be developed.

There are a number of potential limitations to this study. First, this was a cross-sectional study. Many variables were measured at a single time point and may be subject to conditions at the time of measurement. In addition, the study subjects were limited to the population of 66-year-olds. However, the study used a large data set consisting of 103,763 subjects from the medical examination conducted by NHIC, which represents all of Korea. Thus, the findings in the study may be extrapolated to all MetS patients in Korea.

## Conclusion

This study was performed using data from 103,763 older adult individuals (66 years old) with MetS to develop gender-specific management strategies for MetS. Gender differences in older adults with MetS in Korea differ from those of other countries. A higher percentage of females with MetS had abdominal obesity and low HDL-C levels than males, while a higher percentage of males with MetS had elevated BS than females. The prevalence of elevated BP, TG levels, and BS levels was high for both genders. TG levels could be used as a sensitive marker of MetS in Koreans. In addition, females had more combinations of MetS components than did males. Gender differences were also observed for other risk factors for CVD. Combined, these results indicate that attention to gender differences must be paid by both medical staff and health policymakers regarding components of MetS. Based on the results of this study and other similar studies, public health policies and gender-specific strategies focused on older adult individuals with MetS for the prevention of CVD should be formulated.

## Abbreviations

AHA: American Heart Association
BMI: body mass index
BP: blood pressure
BS: blood sugar
CVD: cardiovascular disease
HDL-C: high-density lipoprotein cholesterol
IFG: impaired fasting glucose
IGT: impaired glucose tolerance
MetS: metabolic syndrome
NCEP: National Cholesterol Education Program
NHIC: National Health Insurance Corporation
NHLBI: National Heart Lung, and Blood Institute
TG: triglycerides
VAT: visceral abdominal fat
WC: waist circumference
