# Erratum to: Gender differences in metabolic syndrome components among the Korean 66-year-old population with metabolic syndrome

**DOI:** 10.1186/s12877-016-0241-2

**Published:** 2016-03-24

**Authors:** Sangjin Lee, Young Ko, Chanyeong Kwak, Eun-shil Yim

**Affiliations:** Office for Planning and Coordination, Division of Planning and Coordination, Ministry of Health & Welfare, 13, Doum 4-ro, Sejong-si, 339-012 Republic of Korea; College of Nursing, Gachon University, 191 HambakmoeiroYeonsu-Gu, Incheon, 406-799 Republic of Korea; School of Nursing, Hallym University, 1 Hallymdaehak-gil, Chuncheon, Gangwon-do 200-702 South Korea; Department of Nursing, Daegu Health College, Youngsong-roBuk-gu, Daegu, 702-722 Republic of Korea

## Erratum

Unfortunately, the original version of this article [1] contained an error. The name of the author Chanyeong Kwak was incorrectly spelt as Changyeong Kwak. The correct spelling is included here, and has been updated in the original article.
